# Optimising outcomes of exercise and corticosteroid injection in patients with subacromial pain (impingement) syndrome: a factorial randomised trial

**DOI:** 10.1136/bjsports-2019-101268

**Published:** 2020-08-19

**Authors:** Edward Roddy, Reuben O Ogollah, Raymond Oppong, Irena Zwierska, Praveen Datta, Alison Hall, Elaine Hay, Sue Jackson, Sue Jowett, Martyn Lewis, Julie Shufflebotham, Kay Stevenson, Danielle A van der Windt, Julie Young, Nadine E Foster

**Affiliations:** 1 Primary Care Centre Versus Arthritis, Keele University, Keele, Staffordshire, UK; 2 Haywood Academic Rheumatology Centre, Midlands Partnership NHS Foundation Trust, Stoke-on-Trent, UK; 3 Keele Clinical Trials Unit, Keele University, Keele, Staffordshire, UK; 4 Health Economics Unit, Institute for Applied Health Research, University of Birmingham, Birmingham, UK; 5 Radiology, University Hospitals of North Midlands NHS Trust, Stoke-on-Trent, Staffordshire, UK; 6 Bradwell Hospital, Midlands Partnership NHS Foundation Trust, Stoke-on-Trent, UK

**Keywords:** shoulder, physiotherapy, exercise, steroids, ultrasound

## Abstract

**Objectives:**

To compare the clinical effectiveness of (1) physiotherapist-led exercise versus an exercise leaflet, and (2) ultrasound-guided subacromial corticosteroid injection versus unguided injection for pain and function in subacromial pain (formerly impingement) syndrome (SAPS).

**Methods:**

This was a single-blind 2×2 factorial randomised trial. Adults with SAPS were randomised equally to one of four treatment groups: (1) ultrasound-guided corticosteroid injection and physiotherapist-led exercise, (2) ultrasound-guided corticosteroid injection and an exercise leaflet, (3) unguided corticosteroid injection and physiotherapist-led exercise and (4) unguided corticosteroid injection and an exercise leaflet. The primary outcome was the Shoulder Pain and Disability Index (SPADI), collected at 6 weeks, 6 and 12 months and compared at 6 weeks for the injection interventions and 6 months for the exercise interventions by intention to treat.

**Results:**

We recruited 256 participants (64 treatment per group). Response rates for the primary outcome were 94% at 6 weeks, 88% at 6 months and 80% at 12 months. Greater improvement in total SPADI score was seen with physiotherapist-led exercise than with the exercise leaflet at 6 months (adjusted mean difference −8.23; 95% CI −14.14 to -2.32). There were no significant differences between the injection groups at 6 weeks (−2.04; −7.29 to 3.22), 6 months (−2.36; −8.16 to 3.44) or 12 months (1.59; −5.54 to 8.72).

**Conclusions:**

In patients with SAPS, physiotherapist-led exercise leads to greater improvements in pain and function than an exercise leaflet. Ultrasound guidance confers no additional benefit over unguided corticosteroid injection.

**Trial registration number:**

ISRCTN42399123.

## Introduction

Shoulder pain is an important cause of disability, affecting one in five adults.^1 2^ Half of cases are due to subacromial pain (formerly impingement) syndrome (SAPS).^2^ Treatment guidelines recommend non-surgical management as the mainstay including education/self-management advice, exercise, corticosteroid injection and analgesia.^3 4^

Exercise is thought to reduce SAPS-related pain and disability by addressing posture, muscle (including rotator cuff) weakness, scapular stability and impaired scapulohumeral rhythm, but trials are often small, of poor methodological quality and include only short-term follow-up.^5–9^ Supervised, progressed shoulder exercises are effective in the short term but the optimum type, intensity and duration are unknown.^3 6–8 10–12^ A systematic review of contextual factors associated with outcome concluded that physiotherapists should be involved in the design and delivery of exercise programme for SAPS.^11^ SAPS is commonly treated with corticosteroid injections although their efficacy is debated.^13–15^ Poor response to injection has been attributed to inaccurate placement of the injection,^16^ among other factors. Ultrasound guidance might improve accuracy, but small randomised trials have not consistently confirmed the superiority of ultrasound-guided injection over unguided injection in patients with SAPS.^17–21^

The SUbacromial imPingement syndrome and Pain: a randomised controlled trial Of exeRcise and injecTion trial aimed to assess whether greater improvements in pain and function were obtained with (1) a physiotherapist led, individualised, supervised and progressed exercise programme versus a standardised advice and exercise leaflet, and (2) ultrasound-guided subacromial corticosteroid injection versus unguided injection.

## Methods

### Study design

This was a pragmatic 2×2 factorial single-blind randomised trial conducted within the National Health Service (NHS). The trial protocol has been published previously.^22^ The economic evaluation conducted alongside the trial will be published separately.

### Participants

Participants were referred by their general practitioner (GP) to one of two NHS community musculoskeletal services in Staffordshire, England. Following clinician review of their GP's referral letter, consecutive shoulder pain patients were mailed a participant information sheet prior to a clinical appointment where eligibility was assessed by rheumatologists, rehabilitation medicine specialists, extended scope physiotherapists or GPs with a special musculoskeletal interest. Participants were aged ≥18 years, had no significant shoulder trauma and had a clinical diagnosis of SAPS (pain in the deltoid insertion area, positive Neer or Hawkins-Kennedy tests, and pain on shoulder abduction as was widely accepted at trial commencement).^22 23^ Exclusion criteria were (1) the main complaint being due to neck problems, acromioclavicular pathology, other shoulder disorders, inflammatory arthritis, polymyalgia rheumatica or malignancy; (2) previous (or awaited) ipsilateral shoulder surgery; (3) coagulopathy, warfarin therapy; (4) shoulder injection or shoulder-focused exercise programme in the preceding month and (5) inability to provide informed consent, complete written questionnaires or read documentation written in English. Written informed consent to participate was obtained by a research nurse.

### Randomisation and masking

Participants were randomly allocated in a 1:1:1:1 ratio to one of four treatment groups using stratified block randomisation (stratified by clinic):

1. Ultrasound-guided subacromial corticosteroid injection and physiotherapist-led individualised, supervised and progressed exercise.
2. Ultrasound-guided subacromial corticosteroid injection and an advice and exercise leaflet.
3. Unguided subacromial corticosteroid injection and physiotherapist-led individualised, supervised and progressed exercise.
4. Unguided subacromial corticosteroid injection and advice and exercise leaflet.

A trial administrator in the clinic arranged randomisation through Keele University Clinical Trial Unit’s telephone randomisation service. Participants and clinicians were aware of treatment allocation. The research nurse remained blind to allocation. Participants’ GPs were informed by letter of their patient’s participation.

### Interventions

#### Physiotherapist-led individualised, supervised and progressed exercise

The protocol for the physiotherapist-led exercise programme can be found at: https://www.keele.ac.uk/media/keeleuniversity/ri/primarycare/docs/SUPPORT_Physiotherapy_Intervention_Manual_v3.0_04_01_11_Internet_Version.pdf. The exercise programme was delivered by 20 community-based, musculoskeletal physiotherapists who completed a 2-day training workshop about the exercise treatment protocol. It commenced within 3 weeks of recruitment and injection. Exercise type and dose were individualised, supervised and progressed in 6–8 sessions over a period of 12–16 weeks. Exercises were progressed through three stages guided by an individualised written exercise sheet and a computerised package,^24^ aiming to support the patient back to their specific everyday physical, sporting and occupational activities^25^:

1. Scapular stability exercise and active movement without resistance.
2. Range of movement exercises, isometrics and stretches, with scapular control in pain-free range.
3. Through-range resistance exercises, progressed to encourage rotator cuff muscle strengthening through all ranges of movement.

Treatment approaches included patient self-monitoring, goal setting and a written individualised home exercise programme. Non-attenders were offered up to two further appointments.

#### Advice and exercise leaflet

The leaflet was provided following corticosteroid injection and included information about shoulder anatomy and SAPS; simple self-help messages about analgesia, cold packs and activities; and six standardised specific strengthening and range of motion exercises to be performed 2–3 times daily, with no instructions for individualisation or progression.^3^

#### Ultrasound-guided subacromial corticosteroid injection

Ultrasound-guided injections were performed by one of nine clinicians using the LOGIQ e system with a 12 MHz transducer.^22^ Clinicians either had extensive clinical experience performing ultrasound-guided injections or completed an accredited course on ultrasound-guided subacromial injections; all attended a half-day injection protocol workshop and passed a clinical competency test by a consultant musculoskeletal sonographer (AH). The skin and transducer were cleaned with chlorhexidine 0.5% solution and sterile gel applied to the transducer. The participant sat with the shoulder internally rotated and the ipsilateral hand on the buttock to maximise visibility of and access to the subacromial bursa. The transducer was placed anterolaterally, the hypoechoic subacromial bursa visualised, and a 21 G needle inserted under real-time ultrasound guidance until the needle-tip entered the bursa. A commercially available premixed solution of methylprednisolone 40 mg and 1 mL 1% lidocaine was injected into the bursa.

#### Unguided subacromial corticosteroid injection

Unguided subacromial injections were performed by one of eight clinicians, different to those performing ultrasound-guided injections. Clinicians had extensive clinical experience performing subacromial injections, and attended a half-day injection protocol workshop. The participant sat with their arm hanging with the elbow bent and forearm resting on their lap. The skin was cleaned with chlorhexidine solution 0.5%. A 21 G needle was inserted through the deltoid under the acromion process laterally. The same premixed solution of methylprednisolone 40 mg and 1 mL 1% lidocaine was injected.

Following ultrasound-guided or unguided injection, participants were advised not to drive immediately after injection and to avoid pushing/pulling movements with the affected arm and heavy/repetitive tasks for 2 weeks. A second injection as per treatment allocation was permitted at the treating clinician’s discretion.

### Data collection

Baseline data were collected by self-complete questionnaire immediately before randomisation. Participants were contacted by a blinded research nurse 1-week postrandomisation by text message or telephone (according to patient preference) to assess presence and intensity of shoulder pain (0–10 Numeric Rating Scale (NRS)). All outcome measures were collected at 6 weeks, 6 months and 12 months postrandomisation by postal self-complete questionnaire (adverse events were collected at 6 weeks only). Postal reminders were sent to non-responders 2 and 4 weeks after mailing. Non-responders to the reminders were telephoned by the blinded research nurse to collect the primary outcome measure. Participants unsuccessfully contacted after five telephone attempts were mailed a brief minimum data questionnaire.

### Outcomes

The primary outcome was the Shoulder Pain and Disability Index (SPADI) total score.^26^ Secondary outcomes included the SPADI pain and function subscores, current shoulder pain intensity (0–10 NRS), shoulder pain at night,^27^ patient’s self-reported global impression of change,^28^ Short Form-12,^29^ pain self-efficacy,^30^ fear avoidance,^31^ work (time off, performance, presenteeism),^32^ further corticosteroid injections, treatment preferences and expectations, illness perceptions,^33^ exercise adherence (agreement exercises performed as advised, number of times performed, duration) and treatment satisfaction. Adverse events were collected using clinical case report forms, participant self-report, primary care physician report and the 6 weeks follow-up questionnaire.

### Statistical analysis

#### Sample size

We aimed to detect a small-moderate effect size (standardised mean difference 0.4), equating to an approximately 8-point difference (SD 20) in the SPADI total score for the two main effects (ultrasound-guided vs unguided injection; physiotherapist-led exercise vs leaflet).^34 35^ The primary end point was 6 months for the exercise interventions and 6 weeks for the injection comparisons, since corticosteroid injection improves pain and function more rapidly than exercise.^28 36^ A sample size of 250 participants was needed (80% power; 5% two-tailed significance; 20% lost to follow-up).

#### Analysis

The primary analysis was by intention to treat (ITT) and double-analysed independently by two statisticians, blinded to treatment allocation until the per-protocol analysis. Descriptive statistics (mean (SD) or frequency counts (percentages)) summarised participants’ baseline characteristics by treatment group. We estimated treatment effects using mixed-effect models (linear for numerical outcomes and logistic for dichotomous outcomes) unadjusted and then adjusting for age, gender, baseline SPADI score, pain duration and clinic location. The primary between-group evaluations were the mean differences (adjusted) in total SPADI score at 6 weeks between those randomised to receive ultrasound-guided versus unguided injection and at 6 months between those randomised to physiotherapist-led exercise versus the leaflet. The longitudinal mixed models upheld the full ITT through the inclusion of all randomised participants utilising all available data. Under the missing at random (MAR) assumption (ie, conditional on the observed data such as outcome data at other time points and key baseline covariates, the missingness is independent of the unobserved measurement), the model uses likelihood-based estimation methods to produce unbiased parameter estimates and standard errors. MAR is more plausible than the missing completely at random assumption on which complete case analysis is based.

Secondary analyses included between-group comparisons of the SPADI subscales and other outcome measures at 6 weeks, 6 months and 12 months, and current shoulder pain intensity at week 1. Primary interest focused on main effects (‘at the margins’) evaluation as the two interventions were assumed to act independently of each other. We estimated the interaction effect from a separate regression model (by including an interaction term). A per-protocol sensitivity analysis excluded participants who did not receive the randomly allocated injection or those in the physiotherapist-led exercise group who did not attend 6–8 treatment sessions.^22^ A post hoc complier-average-causal effect (CACE) analysis was also undertaken to measure the unbiased impact of the two interventions across complier subgroups. CACE analysis requires the following assumptions: (1) potential outcomes for each participant are independent of the outcomes for other participants; (2) there is a monotonic relationship between treatment assignment and receipt, that is, there are no individuals for whom assignment to treatment reduces the likelihood of receiving treatment (ie, no defiers); (3) the proportion of compliers taking the active treatment is nonzero; (4) assignment to treatment is random and the proportion of compliers is, on average, the same across intervention and control groups; and (5) exclusion restriction, that is, treatment assignment is independent of the potential outcome given the treatment received. Analyses were performed using Stata V.13 (StataCorp).

### Patient and public involvement

The idea for this trial was developed in collaboration with research users with SAPS who provided feedback on the proposed recruitment and consent processes and choice of trial outcomes. Two patient representatives sat on the independent trial steering committee and provided feedback and advice on the design of questionnaires and participant information leaflets. They also played a full part in monitoring the progress and conduct of the trial.

## Results

**Figure 1:**
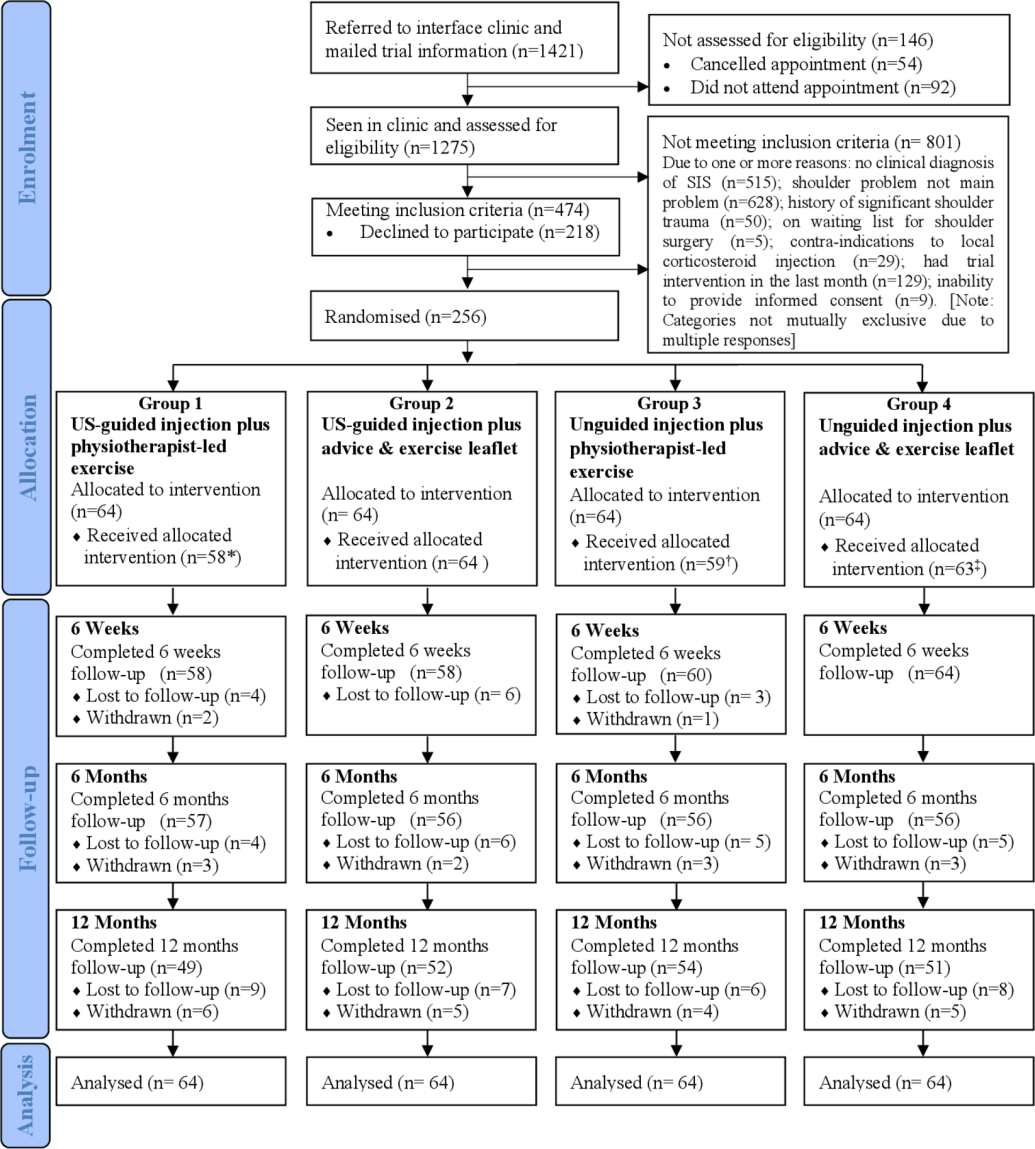
Flow of participants. *6 patients did not attend physiotherapy sessions, one of them also received unguided instead of US-guided injection; †5 patients did not attend physiotherapy sessions, two of them also did not receive unguided injection; ‡1 patient did not receive unguided injection. Reasons for cumulative withdrawal: (a) at 6 weeks– in group 1, time commitments (1), difficulty in completing questionnaires (1), in group 3, relocation (1); (b) at 6 months– in group1, not happy with study arm allocated (1); group 2– exercise did not work (1), relocation (1); group 3- did not want to continue with trial (2); group 4–did not want to continue with the trial (3); (c) at 12 months– in group1, did not want to continue with the trial (1), could not continue completing questionnaires due to frozen shoulders (1), and an operation (1); in group 2–did not want to continue with the trial (2), listed for shoulder surgery (1); in group 3–not happy with the study group allocated (1); in group 4–listed for surgery (1) and not happy with the arm allocated (1). SIS, subacromial impingement syndrome; US, ultrasound.

Between 31 May 2011 and 29 November 2012, 1421 participants were mailed trial information: 1275 were assessed, of whom 474 were eligible (figure 1). A total of 256 eligible participants gave informed consent and were randomised, 64 per group. Demographic characteristics were similar between participants (n=256) and non-participants (n=218): mean (SD) age 53.8 (10.2) vs 55.4 (15.0); 52.0% vs 51.4% female. Participants’ baseline characteristics are shown in table 1. Twelve participants did not receive the allocated intervention and 20 withdrew during follow-up (figure 1). Primary outcome responses were 94% at 6 weeks, 88% at 6 months and 80% at 12 months.

**Table 1:** Baseline characteristics of participants (n=256) by treatment groups

Key characteristics	Overall	US-guided injection plus physiotherapist-led exercise	US-guided injection plus leaflet	Unguided injection plus physiotherapist-led exercise	Unguided injection plus leaflet
	n=256	n=64	n=64	n=64	n=64
Non-clinical characteristics					
Age, mean (SD)	53.8 (10.2)	55.6 (10.5)	54.8 (10.0)	51.9 (10.7)	53.0 (9.5)
Females, n (%)	133 (52.0)	37 (57.8)	29 (45.3)	45 (70.3)	22 (34.4)
Previous or current smokers, n (%)	139 (54.3)	29 (45.3)	38 (59.4)	36 (56.3)	36 (56.3)
Right handed, n (%)	217 (84.8)	59 (92.2)	52 (81.3)	51 (79.7)	55 (85.9)
Currently in paid job, n (%)	141 (55.1)	31 (48.4)	35 (54.5)	34 (53.1)	41 (64.1)
Time off work in past 12 months because of shoulder problem, n (%)	44 (29.3)	10 (28.6)	13 (36.1)	8 (22.9)	13 (29.6)
Shoulder pain interference with work (NRS scale; 0–10) mean (SD)	4.8 (2.9)	4.6 (3.0)	5.2 (2.8)	4.2 (3.0)	4.9 (2.9)
Live alone, n (%)	39 (15.4)	10 (15.9)	12 (18.8)	7 (11.1)	10 (15.9)
Clinical characteristics					
SPADI,* mean (SD)					
Pain subscale score	70.6 (15.5)	69.1 (17.3)	72.9 (14.8)	70.9 (15.6)	69.4 (15.5)
Disability subscale score	55.1 (21.5)	54.3 (22.4)	57.3 (21.1)	57.1 (19.3)	51.6 (22.9)
Total SPADI score	61.1 (18.1)	60.0 (19.2)	63.4 (17.6)	62.4 (16.5)	58.4 (18.9)
Pain severity today (NRS scale; 0–10), mean (SD)	5.7 (2.0)	5.6 (2.1)	5.9 (2.0)	5.4 (1.9)	5.8 (2.0)
Both shoulders affected, n (%)	20 (7.8)	7 (10.9)	3 (4.7)	5 (7.8)	5 (7.8)
Duration of shoulder pain, n (%)					
<3 months	28 (10.9)	6 (9.4)	5 (7.8)	8 (12.5)	9 (14.0)
3–6 months	50 (19.5)	16 (25.0)	13 (20.3)	9 (14.0)	12 (18.8)
6–12 months	74 (28.9)	17 (26.6)	18 (28.1)	26 (40.6)	13 (20.3)
>12 months	104 (40.6)	25 (39.1)	28 (43.8)	21 (32.8)	30 (46.9)
Previous episode of shoulder pain, n (%)	87 (34.0)	16 (25.0)	24 (37.5)	17 (26.6)	30 (46.9)
Troubled by shoulder pain in bed most or every night, n (%)	205 (80.1)	48 (75.0)	51 (79.7)	51 (79.7)	55 (85.9)
Pain self-efficacy scale,† mean (SD)	35.6 (15.3)	35.2 (15.3)	35.4 (13.7)	34.9 (14.0)	36.7 (10.8)
Fear of movement,‡ mean (SD)	27.0 (5.1)	28.0 (5.4)	27.2 (4.8)	26.4 (4.6)	26.4 (5.4)
SF-12-PCS§, mean (SD)	38.1 (9.7)	37.3 (8.9)	38.5 (9.5)	38.1 (10.9)	38.5 (9.8)
SF-12-MCS¶, mean (SD)	47.2 (12.6)	47.9 (12.7)	46.8 (13.3)	45.8 (12.4)	48.1 (12.2)
Other health conditions,** n (%)	137 (53.5)	38 (59.4)	32 (50.0)	37 (57.8)	30 (46.9)
Widespread pain, n (%)	62 (24.8)	15 (23.8)	19 (30.7)	17 (26.6)	11 (18.0)
BMI categories, n (%)					
Normal/underweight	74 (29.5)	17 (26.6)	17 (26.6)	22 (36.1)	18 (29.0)
Overweight	62 (24.7)	22 (34.4)	13 (20.3)	12 (19.7)	15 (24.2)
Obese/morbidly obese	115 (45.8)	25 (39.1)	34 (53.1)	27 (44.3)	29 (46.8)

*Shoulder Pain and Disability Index=Primary outcome measure (each scale/subscale ranges from 0 to 100; 0=no pain/difficulty, 100=worst pain/so difficult it required help).

†10 item scale, score range=0–60 (0=not at all confident, 60=completely confident).

‡Assessed using Tampa scale for kinesiophobia-11– score range from 11 to 44 with higher scores reflecting greater fear of movement or (re)injury.

§SF PCS.

¶SF MCS (scales based on ‘Normalised’ general population average of 50 with SD 10).

**Health conditions include chest problems, heart problems, deafness, problems with eyesight (excluding need for glasses), raised blood pressure, diabetes, stroke, cancer, liver disease, kidney disease and circulation problems in the legs.

BMI, body mass index; MCS, Mental Component Scale; NRS, Numeric Rating Scale; PCS, Physical Component Scale; SF-12, Short Form-12; SPADI, Shoulder Pain and Disability Index; US, ultrasound.

**Figure 2:**
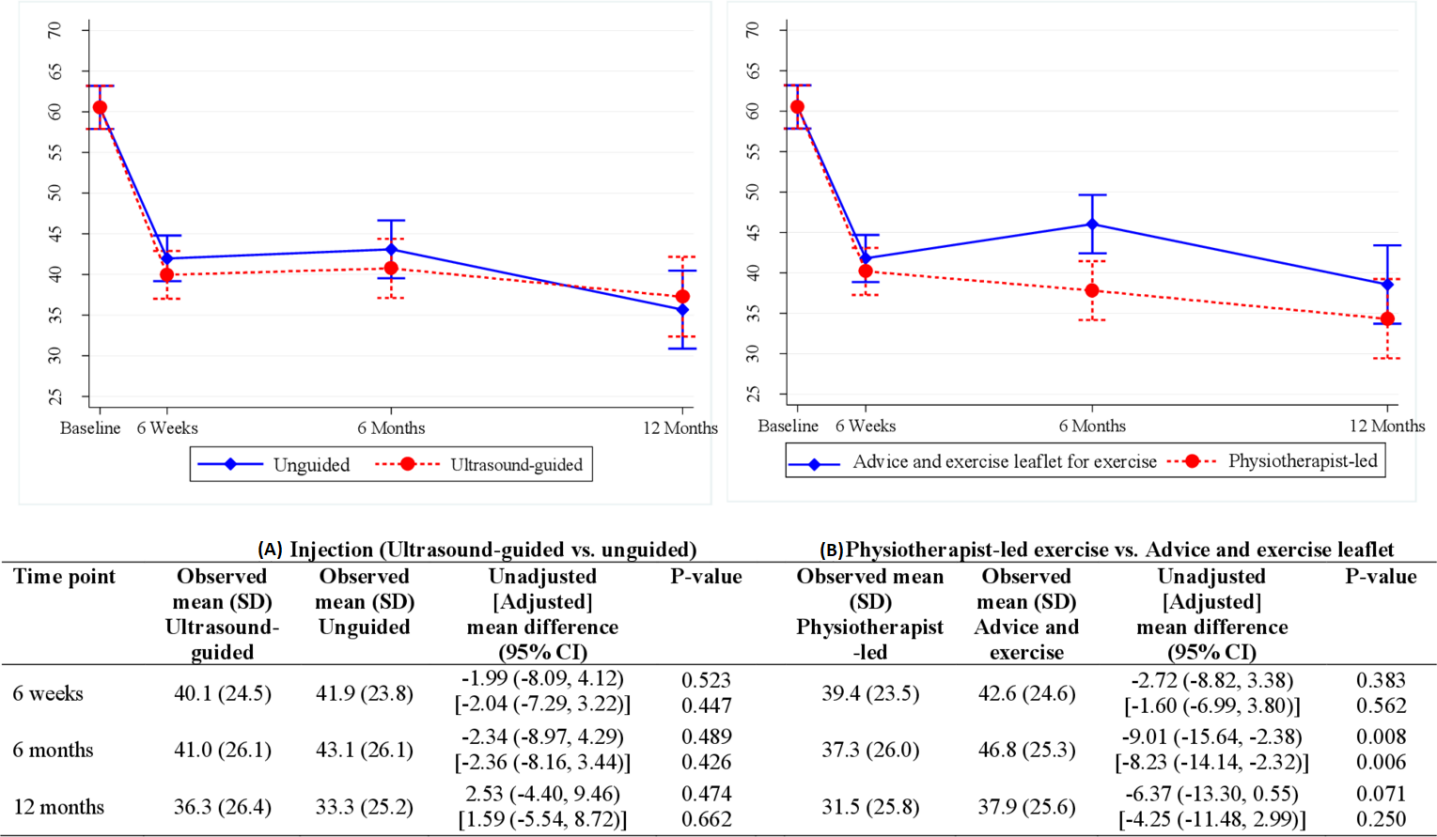
Linear mixed-model derivation of unadjusted and adjusted mean differences with 95% CI for SPADI total score at three follow-up time points for: (A) injection comparison and (B) exercise comparison. The adjusted model was corrected for baseline SPADI scores, age, sex, shoulder problem duration and clinic location. The interaction between the interventions was investigated but was not significant at all time points and hence not presented in the table. The bars in the figure represent the CIs for the adjusted mean, not the differences. SPADI, Shoulder Pain and Disability Index.

Greater improvement in the total SPADI score was seen with physiotherapist-led exercise than the leaflet at 6 months: 6 weeks −1.60 (95% CI −6.99 to 3.80), 6 months −8.23 (−14.14 to –2.32) and 12 months −4.25 (−11.48 to 2.99) (figure 2). There were no significant between-group differences for the two injection groups: 6 weeks −2.04 (95% CI −7.29 to 3.22), 6 months −2.36 (−8.16 to 3.44) and 12 months 1.59 (−5.54 to 8.72).

There were greater improvements in SPADI pain and disability subscales with physiotherapist-led exercise than the leaflet at 6 months but not at 6 weeks and 12 months (table 2). Fewer people in the physiotherapist-led exercise group than the leaflet group reported shoulder pain at night at 12 months. Work performance was worse in the leaflet group than the physiotherapist-led exercise group at 6 months but there were no significant differences in days taken off work or presenteeism. Compared with the leaflet group, those in the physiotherapist-led exercise group perceived that shoulder pain would continue for a shorter duration (timeline) at 6 weeks, had less effect emotionally (emotional representation) at 6 months and on their life (consequences) at 12 months, can be helped by treatments to a greater extent (treatment control) at 12 months, and had greater control over shoulder pain (personal control) at 12 months. Those in the ultrasound-guided injection group perceived that their shoulder pain had less effect emotionally (emotional representation) at 6 weeks and reported lower work presenteeism at 12 months (table 2) than those in the unguided injection group. There were no other significant differences in secondary outcomes between the exercise or injection groups.

**Table 2:** Results for secondary outcomes at 6 weeks, 6 months and 12 months (intention to treat) based on linear mixed-effect model, adjusting for baseline scores, age, gender, duration of pain and location of clinic

Outcome measure	Time point	Injection intervention group				Exercise intervention group			
		US guided	Unguided	Adjusted* between group comparison (reference: unguided)		Physiotherapist led	Advice and exercise leaflet	Adjusted* between group comparison (reference: leaflet)	
Numerical measures		Observed mean (SD)	Observed mean (SD)	Mean difference (95% CI)	P value	Observed mean (SD)	Observed mean (SD)	Mean difference (95% CI)	P value
SPADI† pain subscale	6 weeks	47.6 (25.6)	49.5 (26.0)	−1.92 (−7.68 to 3.83)	0.513	45.6 (25.6)	51.4 (25.7)	−3.03 (−8.96 to 2.89)	0.315
	6 months	47.0 (26.8)	50.1 (28.0)	−3.60 (−9.94 to 2.74)	0.266	42.9 (27.6)	54.3 (26.1)	−9.25 (−15.74 to −2.75)	0.005
	12 months	40.8 (28.7)	40.4 (27.7)	−0.84 (−8.67 to 7.00)	0.834	36.5 (28.0)	44.7 (27.9)	−4.87 (−12.86 to 3.11)	0.231
SPADI† disability subscale	6 weeks	35.6 (25.3)	37.3 (24.2)	−2.47 (−7.79 to 2.85)	0.363	35.7 (23.9)	37.0 (25.4)	−0.21 (−5.68 to 5.27)	0.941
	6 months	37.3 (26.6)	38.6 (26.4)	−1.82 (−7.63 to 4.00)	0.540	33.8 (26.0)	42.2 (26.2)	−7.15 (−13.10 to −1.19)	0.019
	12 months	33.4 (25.8)	29.0 (25.2)	3.09 (−3.93 to 10.12)	0.388	28.5 (25.6)	33.7 (25.4)	−2.98 (−10.13 to 4.18)	0.415
Current shoulder pain severity	1 week	4.9 (2.2)	5.0 (2.3)	−0.15 (−0.73 to 0.44)	0.620	4.9 (2.3)	5.0 (2.2)	0.12 (−0.48 to 0.72)	0.697
	6 weeks	3.6 (2.6)	4.2 (2.7)	−0.58 (−1.23 to 0.07)	0.083	3.4 (2.4)	4.4 (2.8)	−0.63 (−1.31 to 0.05)	0.071
	6 months	3.9 (2.8)	4.4 (3.1)	−0.54 (−1.28 to 0.20)	0.155	3.7 (2.9)	4.6 (2.8)	−0.54 (−1.31 to 0.23)	0.172
	12 months	3.3 (2.6)	3.1 (2.8)	−0.29 (−1.16 to 0.57)	0.506	2.6 (2.7)	3.8 (2.6)	−0.50 (−1.40 to 0.39)	0.271
Pain self-efficacy‡	6 weeks	42.4 (15.1)	39.2 (14.9)	2.74 (−0.04 to 5.51)	0.053	41.5 (14.5)	40.0 (15.6)	1.10 (−1.75 to 3.95)	0.451
	6 months	39.6 (15.7)	40.5 (14.7)	−0.83 (−4.44 to 2.78)	0.652	40.5 (15.5)	39.6 (15.0)	0.67 (−3.07 to 4.41)	0.725
	12 months	43.0 (16.5)	43.6 (15.6)	2.10 (−2.66 to 6.88)	0.387	45.4 (16.5)	41.2 (15.2)	2.79 (−2.13 to 7.71)	0.267
Fear of movement§	6 weeks	24.7 (6.7)	25.2 (6.1)	−1.01 (−2.37 to 0.35)	0.146	24.4 (6.9)	25.5 (5.7)	−0.72 (−2.11 to 0.67)	0.310
	6 months	24.9 (7.1)	25.2 (6.5)	−0.73 (−2.42 to 0.95)	0.394	24.3 (7.0)	25.7 (6.6)	−1.13 (−2.85 to 0.60)	0.201
	12 months	23.9 (7.3)	24.0 (7.0)	−1.20 (−3.36 to 0.96)	0.276	24.0 (7.5)	23.9 (7.2)	0.14 (−2.07 to 2.36)	0.898
**Illness perception**									
Consequences¶	6 weeks	4.3 (2.9)	4.9 (3.0)	−0.47 (−1.18 to 0.24)	0.197	4.3 (2.7)	4.9 (3.1)	−0.33 (−1.06 to 0.40)	0.378
	6 months	4.4 (3.1)	4.8 (3.2)	−0.12 (−0.91 to 0.68)	0.772	4.1 (3.2)	5.0 (3.1)	−0.77 (−1.59 to 0.06)	0.068
	12 months	3.6 (3.1)	3.3 (3.1)	0.13 (−0.79 to 1.05)	0.776	2.9 (3.0)	4.0 (3.1)	−0.97 (−1.92 to -0.02)	0.045
Timeline**	6 weeks	5.7 (3.2)	6.2 (3.2)	−0.61 (−1.41 to 0.20)	0.139	5.3 (3.0)	6.6 (3.2)	−1.19 (−2.01 to -0.36)	0.005
	6 months	6.8 (3.3)	7.0 (3.4)	−0.25 (−1.20 to 0.70)	0.609	6.2 (3.4)	7.5 (3.2)	−0.79 (−1.78 to 0.19)	0.113
	12 months	5.8 (3.7)	5.8 (3.8)	0.01 (−1.16 to 1.19)	0.981	5.4 (3.8)	6.3 (3.7)	−0.74 (−1.94 to 0.47)	0.232
Personal control††	6 weeks	4.7 (3.0)	4.3 (2.9)	0.18 (−0.57 to 0.94)	0.637	4.3 (2.8)	4.7 (3.1)	−0.32 (−1.10 to 0.47)	0.426
	6 months	4.6 (3.0)	4.6 (3.2)	−0.20 (−1.06 to 0.67)	0.652	4.8 (3.1)	4.3 (3.1)	0.39 (−0.51 to 1.29)	0.396
	12 months	5.0 (3.1)	5.4 (3.2)	−0.25 (−1.29 to 0.79)	0.639	5.8 (3.1)	4.5 (3.0)	1.36 (0.29 to 2.43)	0.013
Treatment control‡‡	6 weeks	6.5 (2.8)	5.6 (3.2)	0.76 (−0.01 to 1.52)	0.053	6.4 (2.8)	5.7 (3.2)	0.41 (−0.38 to 1.20)	0.312
	6 months	6.0 (2.9)	5.4 (3.5)	0.41 (−0.51 to 1.32)	0.385	6.2 (2.9)	5.3 (3.4)	0.80 (−0.14 to 1.75)	0.096
	12 months	6.0 (3.4)	5.8 (3.5)	0.59 (−0.55 to 1.74)	0.309	6.6 (3.2)	5.2 (3.5)	1.16 (0.18 to 2.14)	0.020
Emotional representation§§	6 weeks	3.5 (3.4)	4.8 (3.2)	−0.93 (−1.58 to −0.28)	0.005	3.9 (3.3)	4.4 (3.4)	−0.50 (−1.17 to 0.18)	0.148
	6 months	3.8 (3.4)	4.2 (3.5)	−0.16 (−0.97 to 0.66)	0.707	3.5 (3.4)	4.4 (3.4)	−0.87 (−1.70 to −0.03)	0.043
	12 months	2.9 (3.1)	3.1 (3.1)	0.08 (−0.96 to 1.13)	0.875	2.6 (3.1)	3.4 (3.1)	−0.94 (−0.20 to 0.12)	0.083
**Short Form-12 (SF-12)**									
SF-12-PCS	6 weeks	40.7 (10.7)	42.0 (10.1)	−0.67 (−2.52 to 1.17)	0.476	41.4 (10.6)	41.3 (10.3)	0.62 (−1.30 to 2.54)	0.526
	6 months	40.7 (11.2)	41.9 (10.4)	−1.13 (−3.55 to 1.29)	0.360	42.4 (10.5)	40.2 (11.1)	2.46 (−0.04 to 4.97)	0.054
	12 months	41.7 (10.9)	43.3 (12.2)	−0.03 (−3.22 to 3.17)	0.988	43.4 (11.1)	41.7 (12.1)	1.62 (−1.66 to 4.91)	0.333
SF-12-MCS	6 weeks	50.2 (11.1)	48.3 (12.3)	1.42 (−0.61 to 3.44)	0.171	49.9 (12.0)	48.5 (11.6)	1.62 (−0.47 to 3.71)	0.128
	6 months	49.7 (11.9)	48.1 (11.7)	−0.23 (−2.88 to 2.42)	0.865	49.4 (12.0)	48.4 (11.6)	0.11 (−2.62 to 2.84)	0.939
	12 months	49.2 (13.1)	49.9 (10.9)	−1.16 (−4.65 to 2.32)	0.513	49.3 (11.6)	49.8 (12.4)	−1.43 (−5.00 to 2.14)	0.432
**Work outcomes**									
Work performance	6 weeks	1.6 (2.8)	2.0 (2.8)	−0.40 (−1.11 to 0.28)	0.218	1.5 (2.5)	2.1 (3.0)	−0.60 (−1.38 to 0.03)	0.063
	6 months	1.5 (2.6)	2.0 (2.9)	−0.50 (−1.16 to 0.31)	0.251	1.3 (2.5)	2.2 (2.9)	−0.90 (−1.58 to -0.19)	0.016
	12 months	1.1 (2.2)	1.5 (2.4)	−0.40 (−1.01 to 0.21)	0.214	1.0 (2.1)	1.5 (2.5)	−0.50 (−1.18 to 0.13)	0.105
Work presenteeism	6 weeks	7.5 (9.2)	9.3 (9.7)	−1.8 (−4.14 to 0.61)	0.146	7.0 (9.1)	8.9 (9.6)	−1.90 (−4.10 to 0.65)	0.158
	6 months	6.9 (8.9)	8.7 (9.5)	−1.8 (−4.27 to 0.64)	0.141	6.8 (9.1)	8.8 (9.3)	−2.00 (−4.40 to 0.42)	0.106
	12 months	6.1 (8.7)	9.0 (9.6)	−2.9 (−5.36 to −0.32)	0.029	6.9 (9.1)	8.2 (9.4)	−1.30 (−3.88 to 1.26)	0.313
Time off work (days)	6 weeks	1.7 (7.8)	1.2 (10.6)	0.49 (−2.16 to 2.40)	0.687	0.8 (4.8)	2.1 (12.2)	−1.31 (−4.15 to 0.58)	0.278
	6 months	1.1 (7.8)	2.4 (20.2)	−1.27 (−6.20 to 1.5)	0.534	1.1 (8.4)	2.5 (19.9)	−1.38 (−6.32 to 1.74)	0.501
	12 months	1.0 (4.4)	4.3 (21.7)	−3.30 (−8.7 to 0.09)	0.136	3.3 (20.4)	2.1 (9.5)	1.24 (−2.31 to 6.33)	0.577

Categorical measures		n (%)	n (%)	OR (95% CI)	P value	n (%)	n (%)	OR (95% CI)	P value
Global assessment of overall change									
Completely recovered or much better	6 weeks	39 (37.9)	32 (29.9)	1.79 (0.57 to 5.69)	0.321	40 (38.1)	31 (29.5)	1.46 (0.45 to 4.69)	0.529
	6 months	35 (36.7)	31 (34.1)	1.05 (0.35 to 3.16)	0.926	38 (41.3)	28 (29.5)	1.66 (0.54 to 5.12)	0.374
	12 months	36 (44.4)	41 (49.4)	0.72 (0.15 to 3.53)	0.685	46 (56.8)	31 (37.4)	3.47 (0.65 to 18.65)	0.147
Shoulder pain at night									
Troubled most or every night	6 weeks	45 (43.3)	56 (52.8)	0.54 (0.23 to 1.25)	0.153	44 (41.9)	57 (54.3)	0.65 (0.27 to 1.52)	0.315
	6 months	41 (43.2)	46 (50.6)	0.73 (0.31 to 1.76)	0.488	36 (39.6)	51 (53.7)	0.56 (0.23 to 1.37)	0.204
	12 months	32 (39.5)	25 (30.5)	1.66 (0.61 to 4.53)	0.322	19 (23.8)	38 (45.8)	0.34 (0.12 to 0.95)	0.039

*Adjusted for age, sex, current shoulder problem duration and clinic location.

†SPADI subscales, ranges from 0 to 100; 0=no pain/difficulty, 100=worst pain/so difficult it required help.

‡Pain self-efficacy scale: 10 item scale, score range=0–60 (0=not at all confident, 60=completely confident).

§Tampa scale for kinesiophobia-11–score range from 11 to 44 with higher scores reflecting greater fear of movement or (re)injury.

¶How much does your shoulder pain affect your life? rated on a 0–10 Numeric Rating Scale (NRS), 0=no affect at all, 10=severely affects my life.

**How long do you think your shoulder pain will continue? 0–10 NRS, 0=a very short time, 10=forever.

††How much control do you feel you have over your shoulder problem? 0–10 NRS, 0=absolutely no control, 10=extreme amount of control.

‡‡How much do you think your treatment can help your shoulder problem? 0–10 NRS, 0=not at all, 10=extremely helpful.

§§How much does your shoulder problem affect you emotionally? 0–10 NRS, 0=not at all affected emotionally, 10=extremely affected emotionally; 10SF-12-PCS; SF-12-MCS=SF-12 (scales are based on a ‘Normalised’ general population average of 50 with SD of 10).

MCS, Mental Component Scale; PCS, Physical Component Scale; SPADI, Shoulder Pain and Disability Index; US, ultrasound.

Exercise adherence, defined as performing exercises at least once daily, was more common in the physiotherapist-led exercise group than the leaflet group at 6 weeks (85.6% vs 64.1%) and 6 months (63.2% vs 50.8%) but not at 12 months (48.9% vs 53.2%) (table 3). There were no differences in the proportion undertaking exercise for longer than 10 min at any time point. Exercise adherence, frequency or duration did not differ between the injection groups. Confidence in and satisfaction with treatment was greater with ultrasound-guided than unguided injection, particularly at 6-week follow-up, and with physiotherapist-led exercise than the leaflet at all time points. The cumulative number of participants who underwent repeat injection was 4 by 6 weeks, 34 by 6 months and 45 by 12 months. This did not differ between treatment groups at any time point.

**Table 3:** Results for adherence and satisfaction measures at 6 weeks, 6 months and 12 months

Outcome measure	Time point	Injection intervention group				Exercise intervention group			
		US guided	Unguided	Adjusted* between group comparison (referent group: unguided)		Physiotherapist led	Advice and exercise leaflet	Adjusted* between group comparison (referent group: leaflet)	
		N (%)	N (%)	OR (95% CI)	P value	N (%)	N (%)	OR (95% CI)	P value
**Exercise adherence:** doing exercise as often as advised									
Agree/strongly agree	6 weeks	70 (70.0)	73 (70.9)	0.73 (0.16 to 3.32)	0.686	80 (80.1)	63 (60.6)	7.56 (2.19 to 26.03)	0.001
	6 months	55 (59.1)	53 (60.2)	1.06 (0.30 to 3.73)	0.924	65 (73.0)	43 (46.7)	6.09 (1.89 to 19.57)	0.002
	12 months	37 (46.3)	40 (48.2)	0.94 (0.28 to 3.19)	0.923	40 (49.4)	37 (45.1)	1.35 (0.41 to 4.46)	0.615
**Confidence and satisfaction with treatment**									
Confidence with exercise treatment									
Very or quite confident	6 weeks	60 (58.8)	43 (42.2)	2.78 (1.07 to 7.24)	0.037	69 (68.3)	34 (33.0)	8.30 (2.28 to 30.21)	0.001
	6 months	41 (43.2)	31 (34.8)	1.61 (0.56 to 4.61)	0.372	47 (51.1)	25 (27.2)	4.64 (1.50 to 14.32)	0.008
	12 months	36 (47.4)	36 (45.6)	1.52 (0.31 to 7.41)	0.603	43 (55.8)	29 (37.2)	4.25 (0.79 to 22.90)	0.092
Confidence in recommending treatment received									
Very or quite confident	6 weeks	70 (68.6)	57 (54.8)	2.99 (1.09 to 8.16)	0.033	75 (72.8)	52 (50.5)	4.49 (1.57 to 12.90)	0.005
	6 months	58 (60.4)	47 (53.4)	1.53 (0.56 to 4.13)	0.404	57 (62.0)	48 (52.2)	1.84 (0.65 to 5.25)	0.251
	12 months	56 (70.9)	42 (51.2)	5.56 (1.73 to 17.84)	0.004	57 (71.3)	41 (50.6)	3.50 (1.13 to 10.85)	0.030
Satisfaction with treatment									
Very or quite satisfied	6 weeks	75 (74.3)	71 (67.0)	1.89 (0.72 to 4.95)	0.193	93 (88.6)	53 (52.0)	15.81 (5.03 to 49.69)	<0.001
	6 months	63 (65.0)	59 (65.6)	0.79 (0.32 to 1.98)	0.618	75 (81.5)	47 (49.5)	8.86 (3.14 to 25.02)	<0.001
	12 months	42 (55.3)	44 (55.0)	1.11 (0.43 to 2.81)	0.833	46 (59.0)	40 (51.3)	1.40 (0.54 to 3.63)	0.489
Satisfaction with the information received concerning shoulder problem									
Very or quite satisfied	6 weeks	87 (85.3)	85 (79.4)	2.08 (0.59 to 7.39)	0.256	94 (89.5)	78 (75.0)	4.12 (1.03 to 16.29)	0.044
	6 months	78 (81.3)	72 (80.0)	1.09 (0.32 to 3.69)	0.894	81 (88.0)	69 (73.4)	4.86 (1.23 to 19.24)	0.024
	12 months	67 (83.8)	60 (73.2)	2.35 (0.65 to 8.59)	0.195	71 (88.8)	56 (68.3)	8.25 (1.92 to 35.31)	0.004
Extent to which the expectations for shoulder pain relief have been met									
Definitely met	6 weeks	29 (28.4)	24 (22.4)	1.40 (0.51 to 3.85)	0.511	33 (31.4)	20 (19.2)	2.04 (0.72 to 5.75)	0.179
	6 months	26 (27.1)	17 (18.9)	2.22 (0.71 to 6.89)	0.169	30 (33.0)	13 (13.7)	4.52 (1.37 to 14.88)	0.013
	12 months	20 (25.0)	22 (26.8)	0.98 (0.31 to 3.09)	0.974	26 (32.5)	16 (19.5)	1.41 (0.44 to 4.51)	0.562

		Mean (SD)	Mean (SD)	Mean difference (95% CI)	P value	Mean (SD)	Mean (SD)	Mean difference (95% CI)	P value
Overall rating for the results of treatment/care*	6 weeks	7.1 (2.7)	6.1 (3.0)	0.99 (0.25 to 1.74)	0.009	7.3 (2.6)	5.9 (2.9)	1.16 (0.39 to 1.92)	0.003
	6 months	6.5 (3.0)	5.7 (3.3)	0.12 (−0.62 to 0.86)	0.753	6.9 (2.9)	5.3 (3.2)	0.56 (−0.21 to 1.33)	0.154
	12 months	6.9 (2.6)	6.4 (2.8)	0.34 (−0.44 to 1.12)	0.392	7.6 (2.3)	5.8 (2.8)	0.98 (0.16 to 1.81)	0.019

*Overall results of the treatment or care for shoulder pain rated on a 0–10 numeric rating scale with 0 terrible and 10 excellent.

US, ultrasound.

There was no significant interaction effect of combining US-guided injection and physiotherapist-led exercise (interaction coefficient: 6 weeks 4.63 (95% CI −5.91 to 15.17, p=0.389); 6 months 6.65 (−5.01 to 18.32, p=0.264); 12 months 12.76 (−1.56 to 27.07, p=0.081).

Three participants in the unguided injection group did not receive an injection and one in the ultrasound-guided group received an unguided injection. For the injection comparison, the findings of the per-protocol analysis did not differ from the ITT analysis. Of 128 participants randomised to receive physiotherapist-led exercise, 12 (9%) attended no physiotherapy appointments, 42 (33%) attended 1–5 sessions, 71 (56%) 6–8 sessions as per protocol and 3 (2%) 9–10 sessions (median 6; IQR 3–7). Thirteen (10%) participants in the leaflet group reported seeing a physiotherapist about shoulder pain at least once during the follow-up. The mean between-group per-protocol differences in total SPADI (95% CI)) for the exercise interventions were: 6 weeks −1.55 (−6.57 to 3.48), 6 months −6.91 (−13.08 to –0.74) and 12 months −6.66 (−14.24 to 0.92). The CACE results for the primary endpoints were: 6 weeks mean difference (95% CI) for US-guided versus unguided injection −3.45 (−9.51 to 2.61); 6 months for exercise versus no exercise −11.2 (−19.6 to –2.76).

There was one serious adverse event. A participant randomised to receive ultrasound-guided injection and physiotherapist-led exercise was hospitalised with pyelonephritis. Shoulder pain was temporarily more severe following injection in 49 (48%) participants in the ultrasound-guided group and 51 (49%) in the unguided group. Of these, this lasted longer than 3 days in 17 (35%) and 20 (39%) participants, respectively. Minor adverse events such as discomfort during the injection or local skin changes, presyncope, nausea or flushing following the injection were uncommon (ultrasound-guided injection 16 (13%), unguided injection 17 (13%)). Exacerbation of shoulder pain after performing the exercises was reported by 59 (60%) participants who received physiotherapist-led exercise and 60 (59%) who received the leaflet. This improved within a couple of hours in 22 (37%) and 21 (36%), respectively.

## Discussion

### Summary of findings

This is the largest trial of exercise and injection interventions to treat SAPS. Physiotherapist-led, individualised, supervised and progressed exercise produced greater improvements in pain and function than providing a standard advice and exercise leaflet. The between-group difference at 6 months was no longer significant by 12 months when exercise adherence had reduced. Ultrasound guidance provided no additional benefit over unguided injection other than perceived emotional effect of shoulder pain at 6 weeks and lower work presenteeism at 12 months.

### Interpretation: What this study adds for clinicians who treat shoulder pain

We provide further evidence of medium-term beneficial effects of physiotherapist-led exercise for SAPS adding to previous trials reporting short-term improvements.^6^ While the optimal content and duration of exercise remain uncertain,^7 11^ we demonstrate that an individualised, supervised and progressed exercise programme focusing on scapular stability, range of motion and rotator cuff strengthening, is clinically effective. Potential explanations for its effectiveness include the key characteristics of the programme (individualisation, supervision and progression of exercises by physiotherapists over several sessions coupled with an individualised and progressed home exercise programme); the focus on shoulder stability, movement control and rotator cuff strength; high exercise adherence rates; and/or the contact and attention of the physiotherapist over several face-to-face treatment sessions.^25^ For example, in the physiotherapy-led exercise group, the mean number of physiotherapist sessions attended was six, with 86% reporting performing exercises at least once daily at 6 weeks compared with 64% in the leaflet group. Only 56% of participants received 6–8 sessions as per protocol, yet our results highlight the benefits of physiotherapist-led exercise for SAPS. Few randomised trials have compared physiotherapist-led exercise with self-exercise, finding no difference between treatments, although the trials are small and follow-up short.^7^

Baseline SPADI scores were higher (meaning that patients had more severe pain/disability) than in several other trials in SAPS,^36–38^ most of which were undertaken in community populations. Our participants were referred to an NHS musculoskeletal service, which may have followed non-response to primary care management. Subsequent improvements could reflect regression to the mean, Hawthorne effect or placebo effect, although adjustment for baseline SPADI score and other covariates should minimise between-group biases. The difference in the total SPADI score between exercise groups at 6 months was at the lower range of its minimal important change (MIC) of 8–13 points (mean difference 8.2, effect size 0.45).^34 35^ While a between-group MIC for the SPADI is not known, the between-group difference is consistent with previous trials.^36–38^

The marginal differences seen between injection groups contrasts with a 2013 systematic review which reported greater improvements in pain with ultrasound-guided injection compared with unguided injection in people with shoulder pathology.^17^ In studies included in this review, between-group differences were modest, sample sizes were small, follow-up was short and/or the eligibility criteria for inclusion into the study (and thus corticosteroid injection) were heterogeneous. The clinicians who undertook the injections in our study undertook trial-specific training, demonstrated clinical competency, and had extensive clinical experience or had completed accredited training.^22^ We have previously reported that the majority of ultrasound-guided injections in the trial were placed accurately, demonstrating optimal delivery of the intervention.^39^ However, a previous trial found no difference in shoulder pain and function between a systemic intramuscular (gluteal) corticosteroid injection and ultrasound-guided subacromial injection in patients with rotator cuff disease,^40^ suggesting that accurate placement of corticosteroid injection in the subacromial bursa may be less important than previously suggested.

### Strengths and limitations

Strengths of our trial include the factorial design that allowed us to address two primary research questions, the sample size, length of follow-up and intervention training and protocols. A limitation relevant to both the exercise and injection intervention is the single-blinded design, common in non-pharmacological intervention trials, although sham ultrasound guidance could have facilitated double blinding of the injection interventions. However, not blinding for ultrasound guidance would make it more likely to find a difference favouring ultrasound-guided injection, in contrast to our findings. There was no interaction effect between ultrasound-guided injection and physiotherapist-led exercise although this secondary objective was underpowered and 95% CIs wide.

Our trial registration and published protocol stated we would recruit patients with subacromial impingement syndrome (SIS),^22^ consistent with terminology in use at that time. Here, we adopt the term SAPS, recognising that this or rotator cuff-related shoulder pain is now preferred to SIS.^41 42^ SAPS was diagnosed clinically, rather than requiring imaging, which reflects routine clinical practice and increases the generalisability of our findings, although the clinical tests employed lack sensitivity and specificity.^43 44^ Eligibility criteria included the Hawkins-Kennedy test and pain on abduction which are currently recommended to confirm SAPS,^42^ although others have recently proposed that it is feasible to conduct a clinical assessment of shoulder pain without including such special clinical tests as they cannot localise the anatomical structure(s) causing symptoms and should be considered as pain-provocation tests only.^45^ We assessed only self-reported outcomes but not the effect of exercise on shoulder strength, scapular stability or movement, nor can we determine which aspects of the physiotherapist-led exercise programme contributed most to the benefit observed.

### Conclusion

Physiotherapist-led exercise was more effective than an advice and exercise leaflet for patients with SAPS. We provide evidence for policy-makers, payers and services that outcomes are optimised by supervising, individualising and progressing exercise delivery by physiotherapists rather than offering standard exercise advice to all patients. We found no benefit from augmenting injection with ultrasound-guidance, meaning that patients can be offered injection without requiring specialist skills, training and equipment necessary for ultrasound-guided injection.

**Key messages**

What are the findings?

- Our trial is the largest of exercise and injection interventions for subacromial pain syndrome.
- Physiotherapist-led, individualised, supervised and progressed exercise provides greater improvements in pain and function than providing an exercise leaflet, whereas ultrasound-guided subacromial corticosteroid injection provides no additional clinical benefit over unguided injection.

How might it impact on clinical practice in the future?

- Patients should have access to physiotherapist-led exercise programmes rather than offering the same standard advice and exercise information to all patients.
- Subacromial corticosteroid injections can be performed unguided without guidance from ultrasound.
